# Structural mechanism of human HCN1 hyperpolarization-activated channel inhibition by ivabradine

**DOI:** 10.1016/j.jbc.2024.107798

**Published:** 2024-09-20

**Authors:** Tong Che, Wei Zhang, Xinyu Cheng, Sijia Lv, Minqing Zhang, Yuting Zhang, Tingting Yang, Weiwei Nan, Shuangyan Wan, Bo Zeng, Jian Li, Bing Xiong, Jin Zhang

**Affiliations:** 1 The MOE Basic Research and Innovation Center for the Targeted Therapeutics of Solid Tumors, School of Basic Medical Sciences, Jiangxi Medical College, Nanchang University, Nanchang, Jiangxi, China; 2 The Second Affiliated Hospital, Jiangxi Medical College, Nanchang University, Nanchang, Jiangxi, China; 3 Shenzhen Crystalo Biopharmaceutical Co, Ltd, Shenzhen, Guangdong, China; 4 Key Laboratory of Medical Electrophysiology, Ministry of Education and Sichuan Province and Institute of Cardiovascular Research, Southwest Medical University, Luzhou, Sichuan, China; 5 Department of Endocrinology, Affiliated Hospital of Southwest Medical University, Luzhou, Sichuan, China; 6 College of Pharmacy, Gannan Medical University, Ganzhou, Jiangxi, China; 7 Department of Medicinal Chemistry, Shanghai Institute of Materia Medica, Chinese Academy of Sciences, Shanghai, China

**Keywords:** HCN1 channel, cryo-EM, ivabradine, inhibitor, drug discovery

## Abstract

The hyperpolarization-activated cyclic nucleotide-gated (HCN) channels play a crucial role in regulating neuronal excitability. Despite growing evidence supporting the therapeutic potential of HCN1 inhibition in treating neurological disorders, the structural basis of channel inhibition by inhibitor has remained elusive. Here, we present the cryo-electron microscopy structure of human HCN1 channel in complex with inhibitor ivabradine, the drug on the market that acts on HCN channels. Combining electrophysiology, mutagenesis, and molecular dynamics simulations, our findings reveal that ivabradine binds to a previously unidentified pocket formed between the S4, S1, and HCN domain. Furthermore, through structure-based virtual screening, we identify two Food and Drug Administration-approved drugs that can inhibit the HCN1 channel by interacting with the ivabradine-binding site. Our results not only provide insights into the structural intricacies of ivabradine-mediated inhibition, but also offer a potential pharmacological framework for developing novel drugs targeting the HCN1 channel. The elucidation of these molecular interactions serves as a foundational step in advancing therapeutic strategies for modulating HCN1 activity, contributing to the broader landscape of drug discovery and development in this area.

The hyperpolarization-activated and cyclic nucleotide-gated ion channels comprise four family members (HCN1-4) and are activated by hyperpolarizing potentials (1, 2). These channels are widely expressed in both the heart and nervous system (3, 4). The HCN1 subtype is notably distinguished by its primary localization in both the central and peripheral nervous systems (5). Dysfunction in HCN1 channels can lead to neuronal hyperexcitability and hypersynchronous firing, phenomena associated with various neurological disorders (6, 7). Numerous human mutations linked to epilepsy have been identified in the HCN1 gene, with critical mutations potentially leading to early infantile epileptic encephalopathy (8, 9, 10, 11). Animal studies have also shown that decreased HCN1 expression can induce antidepressant effects and enhance working memory in rats (12, 13). These findings indicate that HCN1 could be a promising target for treating epilepsy (14, 15), depression (6, 16), and cognitive impairments in brain diseases (17, 18).

Ivabradine is the only drug on the market that acts on HCN channels (19). It specifically blocks the pacemaker I_f_ channel of the cardiac sinoatrial node (20), which is mainly composed of HCN4 channel and a small amount of HCN1 channel. Due to its poor ability to penetrate the blood–brain barrier (21), research on its neurological effects is limited. Studies suggest that coadministration of ivabradine with the P-glycoprotein inhibitor elacridar enhances drug permeability into the brain, significantly and persistently reducing absence seizures (22). These indicated the potential of this class of drugs as a novel therapeutic avenue for absence seizures. However, development of potent, selective and brain-penetrant inhibitors of HCN1 is hampered by limited understanding of the molecular mechanism of HCN1 inhibitor (18, 23).

In this study, we explore the molecular basis of human HCN1 (hHCN1) inhibition by ivabradine using cryo-EM, electrophysiology, and molecular dynamics (MD) simulations. Our structure reveals that ivabradine binds to a previously unprecedented pocket formed between the S4, S1, and unique HCN domain. Moreover, using structure-based virtual screening, we have discovered that brain-penetrant haloperidol is capable of inhibiting the HCN1 channel through interaction with the identical binding site. Overall, these findings provide a rational basis for small molecule drug design for the treatment of HCN1-mediated diseases.

## Results

### Inhibition of HCN1 by ivabradine

**Figure 1:**
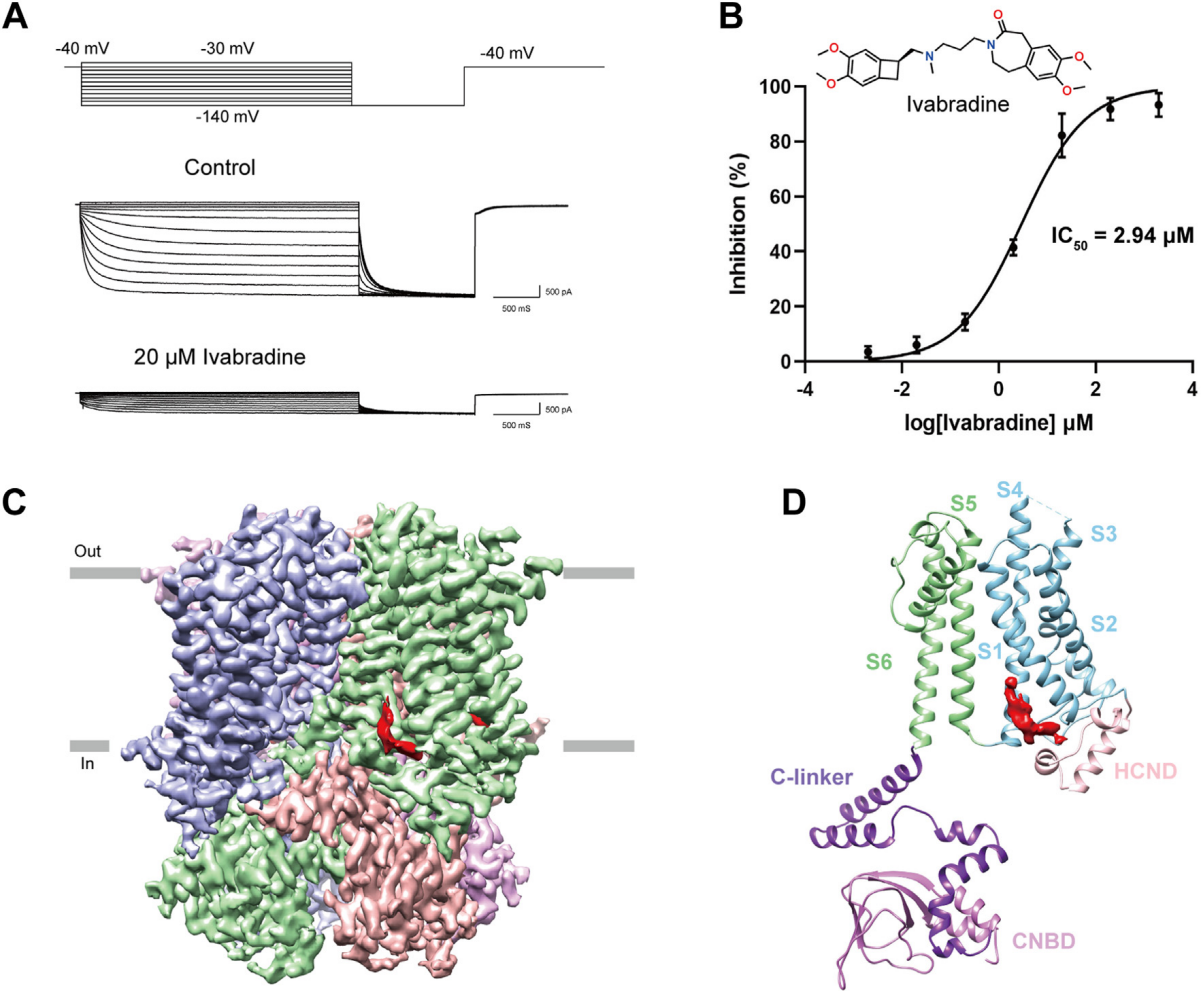
**Functional and cryo-EM characterization of HCN1 inhibition by ivabradine.***A*, the inhibitory effect of ivabradine on WT hHCN1 overexpression, measured by whole-cell patch-clamp electrophysiological recordings. *B*, concentration-response curve for ivabradine inhibition. Data are shown as n = 6 independent experiments. *C*, cryo-EM density maps of ivabradine-bound HCN1 is shown in side view. The *purple*, *green*, *light*, and *dark pink* colors represent each HCN1 subunit, *red* color shows cryo-EM densities for ivabradine. *D*, single subunit ribbon diagram of ivabradine-bound HCN1 structure is shown in side view. *Red* surface shows cryo-EM densities for ivabradine. HCN, hyperpolarization-activated cyclic nucleotide-gated; hHCN1, human HCN1.

The electrophysiological characteristics of WT hHCN1 were recorded using whole-cell patch-clamp. Application of a voltage range from −180 to −30 mV during recordings from HeLa cells transiently transfected with WT hHCN1 revealed the presence of inward hyperpolarization-activated currents, denoted as I_h_ (Fig. S1) (3). Activation of the HCN1 channel occurred in response to membrane hyperpolarization. In the presence of 20 μM ivabradine, I_h_ was significantly reduced (Fig. 1*A*). Subsequent concentration-dependent measurements of I_h_ inhibition at a membrane potential of −140 mV yielded a half-maximal inhibitory concentration (IC_50_) of 2.94 ± 0.61 μM, a value consistent with findings from previous studies (Fig. 1*B*) (24).

### Cryo-EM analysis of HCN1 with ivabradine

To study the binding site of ivabradine in HCN1, we expressed HCN1 structure by genetically engineering an hHCN1 construct with a truncated C terminus (residues 636–865) (25), which was fused with a maltose-binding protein (MBP) tag at the N terminus. The expression of this construct was conducted using the Bac-Bac expression system (Fig. S2). Ivabradine was supplemented to the protein at a final concentration of 1 mM for grid preparation. Micrographs of HCN1-ivabradine showed particles with diverse angular coverage and secondary structure features were readily visible in 2D class averages (Fig. S3*A*). Processing of these data (Fig. S4) resulted in 3D reconstruction of the hHCN1-ivabradine map with a 4-fold rotational symmetry (C4).

Reconstructions of HCN1 in the ivabradine bound states were carried out to an overall resolution of 3.23 Å (Fig. S3, *B* and *C* and Table S1). The overall architecture of ivabradine-bound HCN1 closely resembles the previously characterized apo state (25), with an RMSD of 0.5 Å. Overall, the structures form a symmetric homotetramer (Fig. 1*C*), each subunit comprises six alpha-helical transmembrane domains, with two forming the pore (S5-S6) and four forming the voltage sensor domain (S1-S4) (Fig. 1*D*). The cytoplasmic domain contains an N-terminal conserved HCN domain (HCND) and a C-terminal cyclic nucleotide-binding domain, which is connected to transmembrane helix S6 through a helical C-linker domain (Fig. 1*D*).

### Structure of ivabradine binding sites

**Figure 2:**
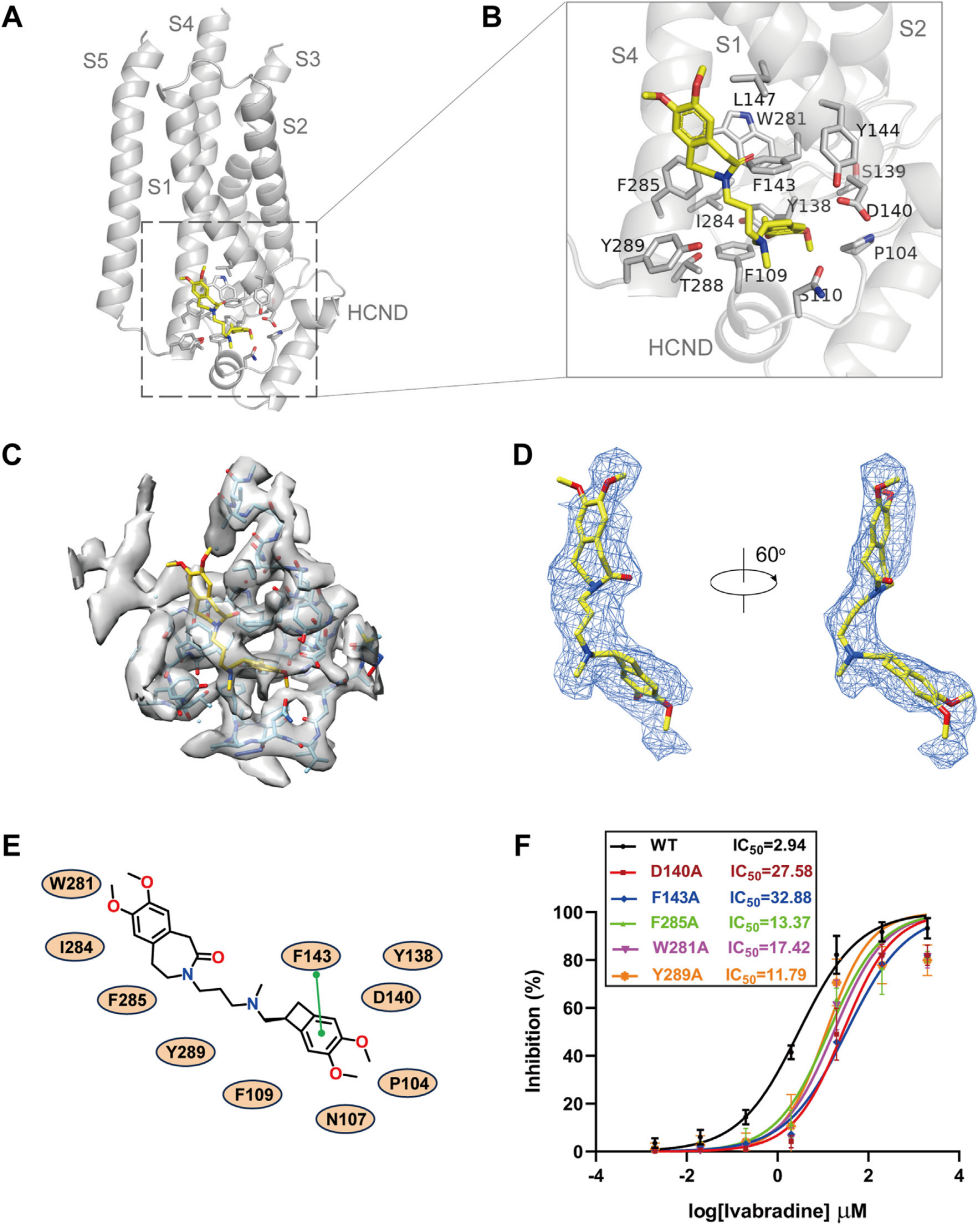
**Structure of the HCN1 channel in complex with ivabradine.***A*, overview of the ivabradine-binding site in HCN1. Ivabradine is shown as *yellow sticks*. *B*, detailed view of the binding site, illustrating the interactions between ivabradine and HCN1. The side chains of crucial residues are shown in *sticks*. *C*, the detailed binding site density map at 3.5σ is shown. *D*, the density is shown in *blue* mesh for ivabradine, which is depicted in sticks. *E*, cartoon representation of the interaction between ivabradine and HCN1. *Green solid lines* represent π-π stacking interaction. *F*, the inhibitory effects of ivabradine on various HCN1 mutants (D140A, F143A, F285A, W281A, and Y289A), measured by electrophysiological recordings. HCN, hyperpolarization-activated cyclic nucleotide-gated.

A nonprotein density is observed, surrounded by the S1, S4, and the unique HCND at the interface between the lipid bilayer and cytoplasmic side, a feature not present in any of the previous HCN1 and HCN4 structures (Figs. 1, *C* and *D*, 2*D*, and S5) (25, 26, 27). This density exhibits a shape and size consistent with the chemical structure of ivabradine (Fig. 1*B*).

Several residues are in close proximity to the ligand density of ivabradine, indicating a direct interaction between the drug and the channel. Specifically, the side chain of F143 on S1 forms π-stacking interactions with the methoxyphenyl ring of ivabradine (Fig. 2, *A–D*). Additionally, N107 and F109 on the HCND, D140 on S1, W281, F285, and Y289 on S4 exhibit hydrophobic and van der Waals interactions with ivabradine. These residues implicated in ivabradine binding are conserved across HCN1-4 (Fig. S6), supporting the nonselective nature of ivabradine with respect to the HCN isoforms.

To elucidate the contribution of these residues to the inhibitory effect of ivabradine, we introduced mutations in ivabradine-interacting residues, converting them into alanines (Fig. 2*F*). Notably, mutations such as D140A and F143A led to a dramatic decrease in ivabradine potency (Fig. 2*F*). These mutational effects align with the structure of the HCN1-ivabardine complex, indicating that residues in the S4 are critical for channel activation and ligand recognition at the interface between the membrane and cytosol.

### MD simulations of ivabradine binding sites

**Figure 3:**
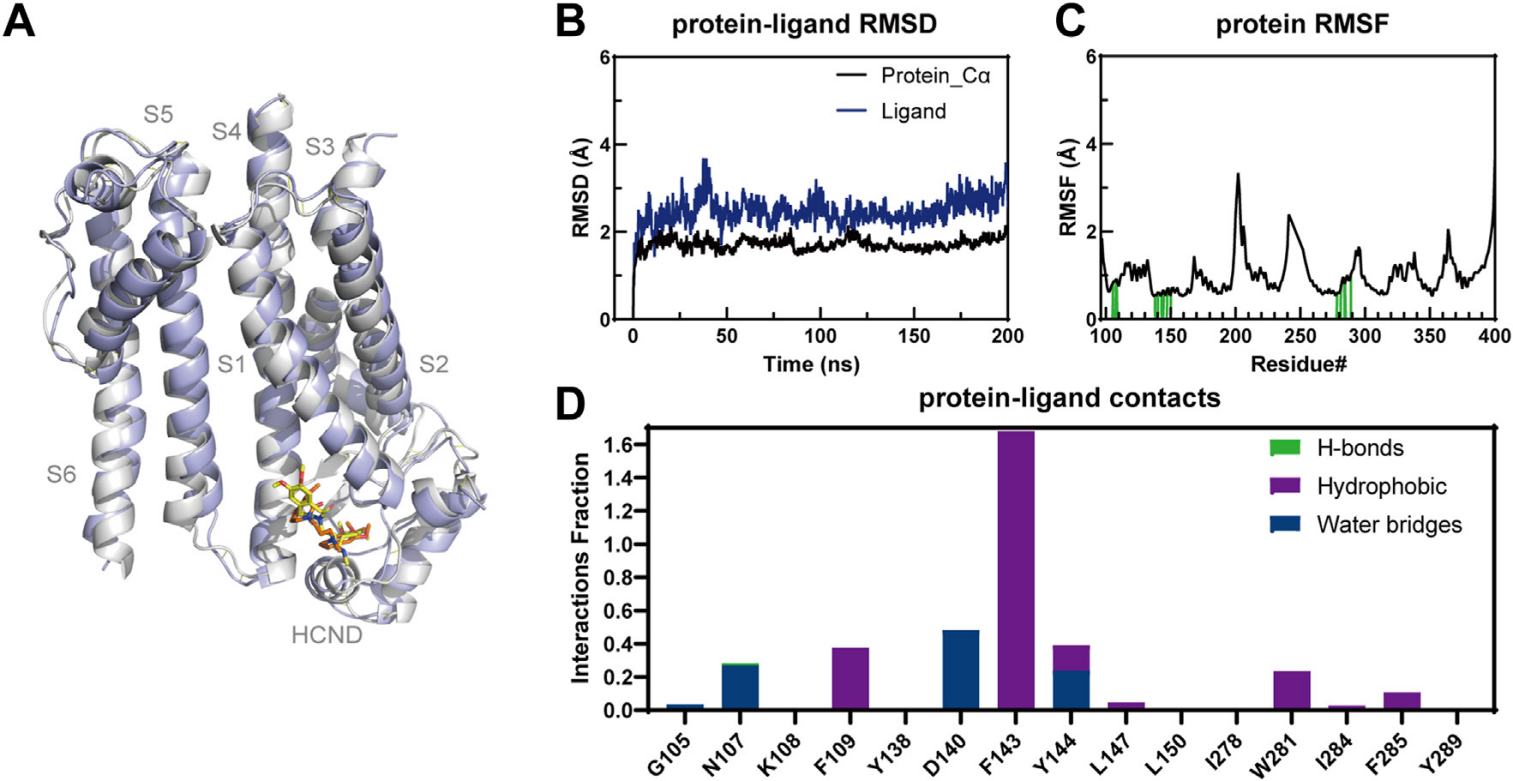
**MD simulations.***A*, representative MD structure (*blue* cartoon and *brownish sticks*), aligned to the cryo-EM structure (*gray* cartoon and *yellow sticks*). *B*, RMSD of ivabradine with HCN1 complex over simulation time. *C*, RMSF values of HCN1 complex residues interacting with ivabradine. Residues that interact with the ligand are marked with *green-colored vertical bars*. *D*, the protein-ligand contacts within the HCN1-ivabradine complex. HCN, hyperpolarization-activated cyclic nucleotide-gated; MD, molecular dynamics; RMSF, root mean square fluctuation.

To further assess the stability of the protein-ligand complex in the ligand-binding pocket of HCN1, we conducted a MD simulation within a lipid bilayer (28). The simulation process for the complex extended over 200 ns. RMSD and root mean square fluctuation were calculated using C-alpha atoms and ligand to examine the stability and conformation of the protein and ligand throughout the MD simulation period. The results indicated that the position of ivabradine in the ligand-binding pocket of HCN1 remained stable from the initiation and throughout the simulation period (Fig. 3, *A*–*C*).

Furthermore, protein interactions with ivabradine were monitored throughout the simulation. These interactions were categorized by type and summarized (Figs. 3*D* and S7). Notably, D140 and F143 exhibited interaction fragments above 0.4, suggesting that over 40% of the simulation time interaction with ivabradine was maintained. F143 residue showed multiple contacts of same subtype with ivabradine (above 1.68). The results showed that ivabradine is stabilized by π-stacking interactions between the methoxyphenyl ring of ivabradine and the phenyl of F143 on S1. Additionally, the binding interface between ivabradine and HCN1 is supported by previous mutagenesis studies (Fig. 2*F*).

### Molecular mechanism of HCN1 inhibition by ivabradine

**Figure 4:**
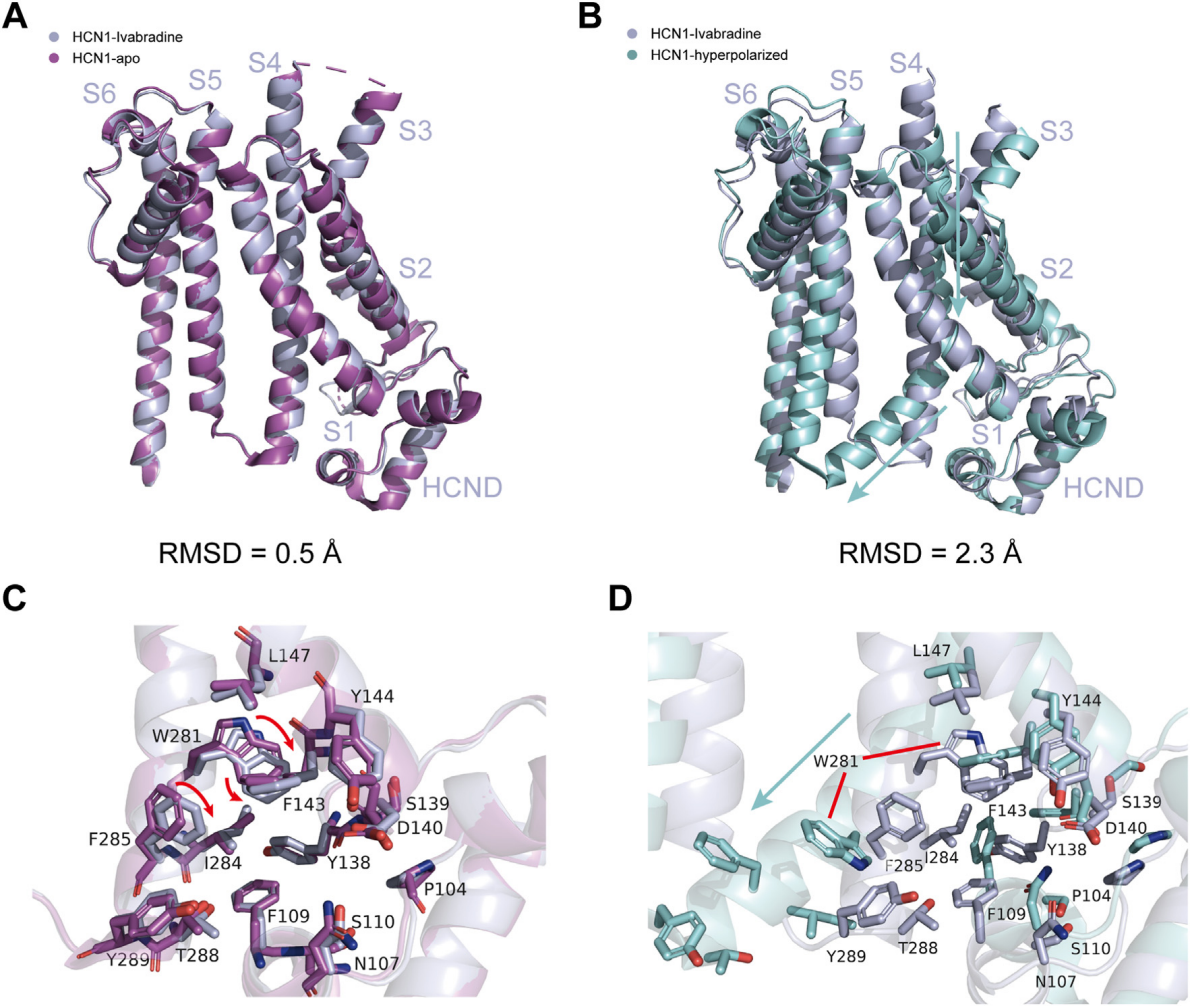
**Structure comparison between apo hHCN1, hyperpolarized hHCN1 and ivabradine-bound hHCN1.***A* and *B*, the overall conformational difference in TMD between apo hHCN1 (*magenta*, PDB: 5U6O), hyperpolarized hHCN1 (*cyan*, PDB: 6UQF) and ivabradine-bound hHCN1 (*light blue*). *C* and *D*, comparison of ivabradine binding site between apo hHCN1 (*magenta*, PDB: 5U6O), hyperpolarized hHCN1 (*cyan*, PDB: 6UQF) and ivabradine-bound hHCN1 (*light blue*). HCN, hyperpolarization-activated cyclic nucleotide-gated; hHCN1, human HCN1; PDB, Protein Data Bank; TMD, transmembrane domain.

The overall structures of HCN1-ivabradine closely resemble those of apo HCN1, with an RMSD of 0.5 Å (Fig. 4*A*). In the ivabradine binding site, the residues F143, W281, and F285 exhibit slight inward conformational changes compared to apo HCN1 (Fig. 4*C*), likely induced by ivabradine binding. Comparing hyperpolarized HCN1 with ivabradine-bound HCN1 reveals that the S4 segment undergoes a downward movement into two sub-helices, leading to a conformational change, with an RMSD of 2.3 Å (Fig. 4*B*). In the ivabradine-bound state, W281 is oriented toward S1, nestled within a hydrophobic cavity at the interface of S4, S1, and the HCND (Fig. 4*D*). Conversely, in the hyperpolarized state, the intracellular S1-S4-S5 region exhibits reduced compactness, and W281 is extricated from the hydrophobic cavity, thereby disrupting interactions with S1 (26, 29). Our investigation establishes that ivabradine induces hydrophobic interactions with W281, thereby stabilizing it within the hydrophobic cavity and maintaining channel closure.

### Structure-based virtual screening

**Figure 5:**
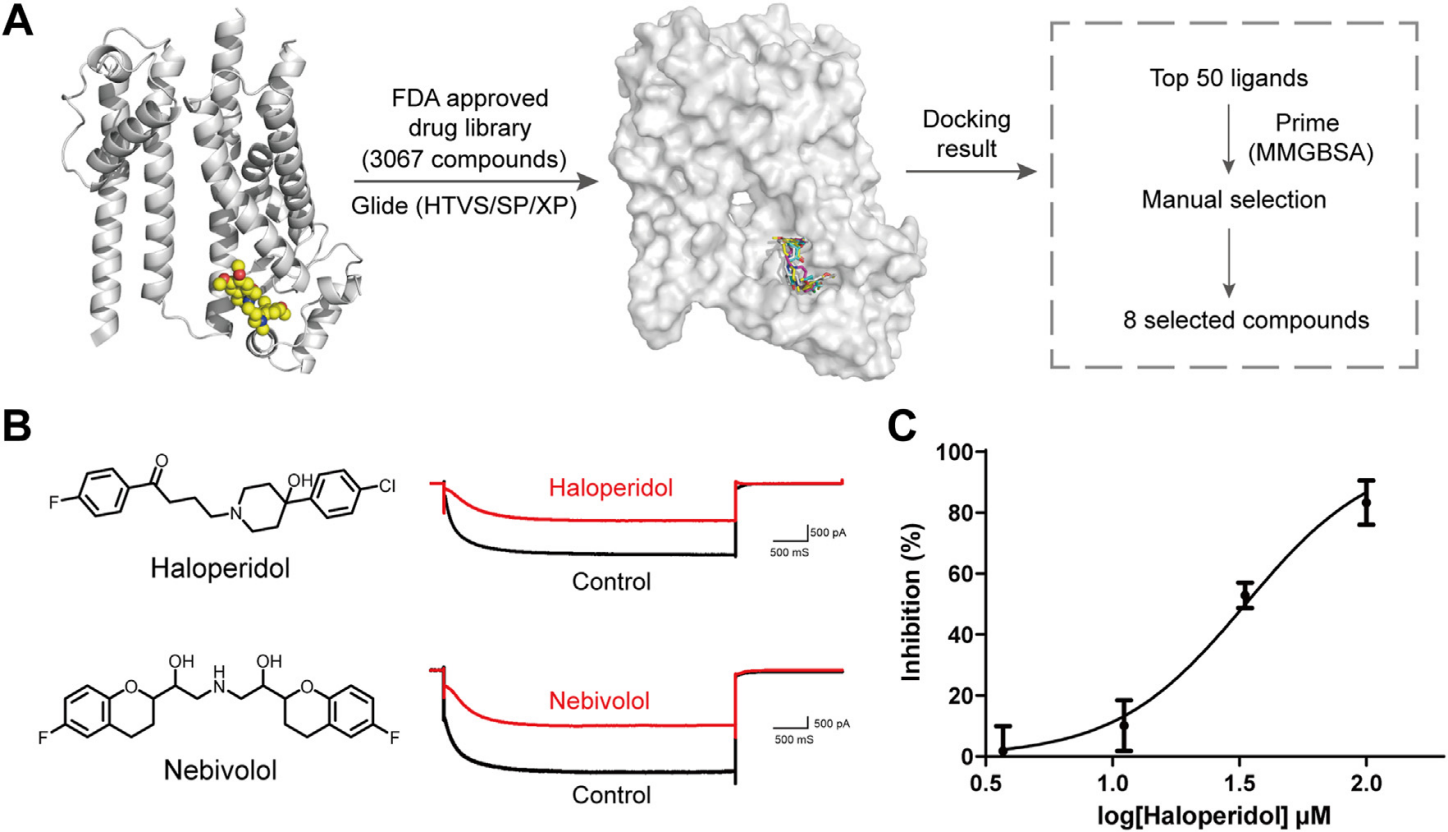
**Structure-based virtual screening for HCN1.***A*, schematic representation of the virtual screening strategy. *B*, representative current and structures of haloperidol and nebivolol. *C*, concentration-response curve for haloperidol inhibition. Data are shown as n = 3 independent experiments. HCN, hyperpolarization-activated cyclic nucleotide-gated.

The defined binding pocket of the HCN1-ivabradine complex, as revealed by the cryo-EM structure, provided an opportunity to explore novel inhibitors with different scaffolds (30, 31). Leveraging the cryo-EM complex structure, we conducted virtual screening *via* molecular docking. The screening initiative involved the Food and Drug Administration drug library (3067 compounds) obtained from Selleckchem (Cat #L1300). Employing a systematic three-step molecular docking approach (from fast-HTVS to slow-XP modes) using the Glide v9.1 program in Schrödinger software (https://www.schrodinger.com/platform/products/glide/) (Fig. 5*A*), we screened the library. Subsequently, the top-ranked 50 compounds from the final docking step underwent further evaluation through Prime MM-GBSA studies. Ultimately, eight compounds were selected and procured for subsequent activity assessment (Fig. S8*A*).

The selected compounds underwent testing through whole-cell patch-clamp recordings. The results suggest that the examined compounds exhibit a measurable inhibitory effect on HCN1, effectively diminishing the current associated with HCN1 hyperactivation (Fig. S8*B*). Notably, haloperidol, being a typical antipsychotic (32), emerged as particularly potent inhibitors of this current (IC_50_ = 33.43 μM), although its efficacy was less pronounced compared to ivabradine (Fig. 5, *B* and *C*). This provides a valuable pharmacological foundation for the development of innovative drugs targeting modulation of the HCN1 channel.

## Discussion

HCN1 channels represent a pivotal class of drug targets with significant clinical implications for disorders such as epilepsy, depression, and cognitive impairments. However, a current challenge in the field is the identification of the ligand-binding pockets within HCN channels, impeding the discovery and development of relevant medications. Our study has made a significant discovery, demonstrating that the drug ivabradine binds to an unprecedented binding pocket within HCN1. This binding pocket includes the HCND region along with a hydrophobic pocket formed by S1 and S4, which differs from the previously hypothesized pore location (33, 34). This discovery implies the existence of multiple ligand-binding sites within HCN channels, similar to other ion channels (35).

**Figure 6:**
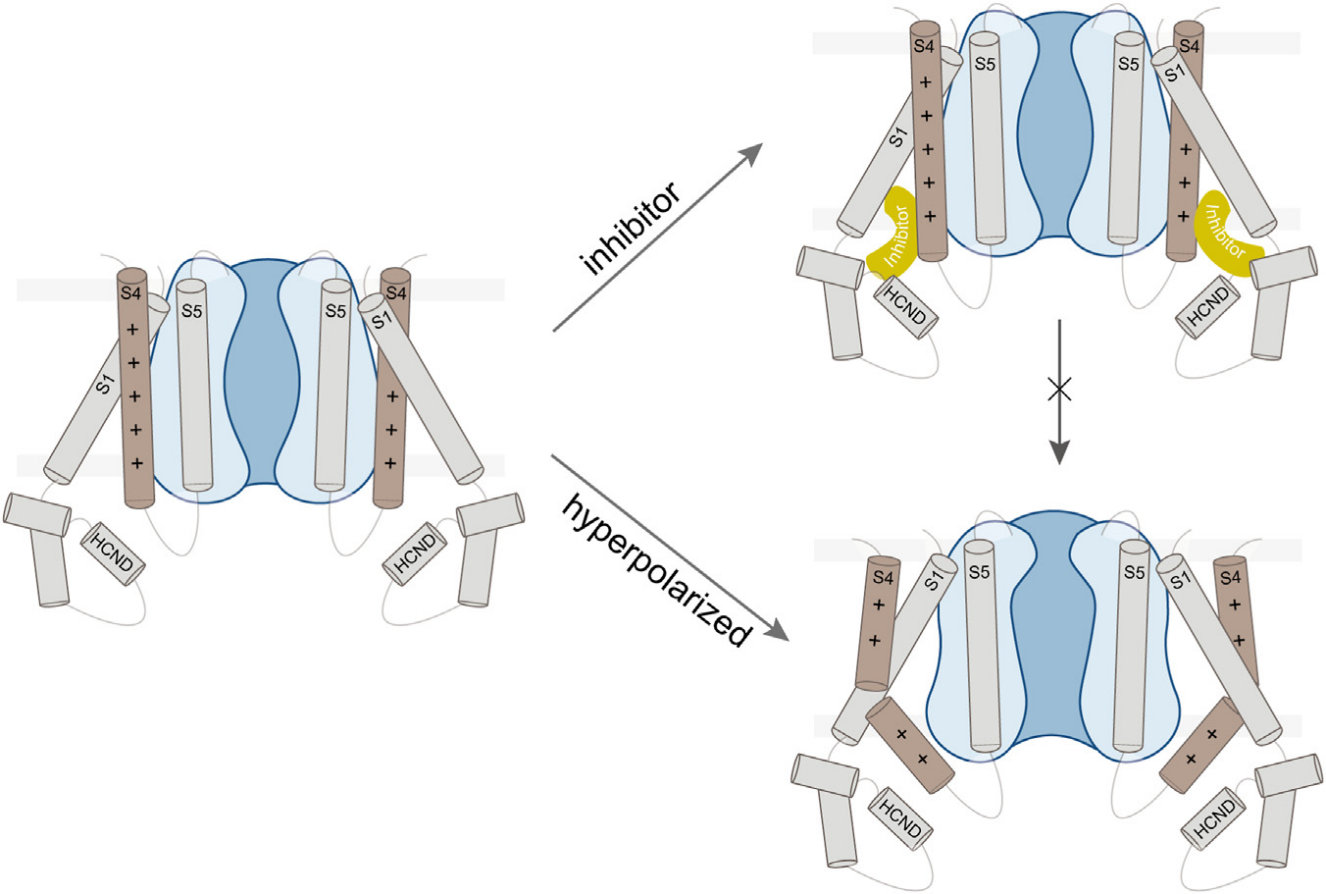
**The mechanism model of HCN1 inhibition by ivabradine.** The inhibitor ivabradine acts like a wedge, binds to the pocket formed by the S4, S1, and HCN domain, thus preventing the conformational change of the S4 helix and maintaining the HCN1 channel in a closed conformation. HCN, hyperpolarization-activated cyclic nucleotide-gated.

The activation mechanism of HCN channels suggests that hyperpolarization triggers a downward movement of S4, leading to its splitting into two subhelices and facilitating channel opening (26, 29, 36). MD simulations analysis highlighted W281 in the lower half of S4 as a potentially critical residue in HCN1 channel gating (37). The inhibitor acts like a wedge, engendering hydrophobic interactions with W281, thus preventing the conformational change of the S4 helix and maintaining the HCN1 channel in a closed conformation (Fig. 6). This finding is consistent with the accurate characterization that ivabradine blocks HCN1 and HCN4 channels through distinct mechanisms: it functions as a “closed-channel blocker” for HCN1 and an “open channel clocker” for HCN4 (24, 38). Furthermore, a recent study also confirmed that ivabradine interacts with HCN4 as an “open channel” blocker, which obstructs the open pore of HCN4 (39).

The clarification of the drug-binding pocket carries significant implications in the field of drug discovery. Considering that HCN1 is the primary isoform expressed in the central and peripheral nervous systems, it stands out as a promising target for addressing neurological disorders. Nevertheless, the restricted blood–brain barrier permeability of ivabradine limits its therapeutic applicability in central nervous system pathologies. Through structure-based virtual screening, we have pinpointed haloperidol with potential HCN1-blocking properties, establishing a groundwork for future exploration of targeted agents for central nervous system intervention.

In summary, our reported structures of HCN1 have unveiled unique binding pockets for ivabradine. Furthermore, through the integration of structure-based virtual screening, a series of drug molecules with novel scaffolds capable of binding to HCN1 have been identified. This further solidifies the distinct structural pocket of ivabradine. Currently, small molecules targeting the HCN channels lack sufficient specificity and exhibit poor selectivity. The clarification of the drug-binding pocket carries significant implications in the field of drug discovery. Our research also lays the structural groundwork for the discovery of molecules with enhanced activity and selectivity within the HCN channels.

## Experimental procedures

### Construct

A DNA segment encoding the truncated hHCN1 channel (residues 636–865) was synthesized and cloned into the pEG BacMam vector. To enhance protein purification, an MBP tag was appended to the N terminus of HCN1 through a linker featuring the HRV 3C cleavage site. For electrophysiological experiments, full-length WT or mutant hHCN1 was cloned into a pcDNA3.1 vector.

### Expression and purification

The MBP-HCN1 protein was expressed and purified based on our previously established protocols (40, 41). Briefly, HEK293S GnTI^−^ cells were transduced with 10% (v/v) P4 baculovirus at a density of 2.0 to 3.0 × 10^6^ cells/ml. Twenty-four hours after transduction, 10 mM sodium butyrate was added to boost protein expression and harvested at 72 h. Prior to solubilization, cells were resuspended for 30 min in a hypotonic lysis buffer (20 mM KCl, 0.5 mM MgCl_2_, 2 mM DTT, 10 mM Tris pH 8.0, and 1%(v/v) EDTA-free protease inhibitor cocktail). The lysate was then homogenized by Dounce homogenizer over 40 times and rolled at 4 °C for 2 h. The lysate was then centrifuged at 39,800*g* for 35 min to sediment the crude membranes, which were then homogenized and extracted by the addition of 0.5% w/v detergent (LMNG-CHS = 5:1). This was then rolled at 4 °C for 3 h, after which the solubilized membranes were clarified by centrifugation at 39,800*g* for 35 min. The supernatant was incubated in MBP beads at 4 °C overnight under gentle agitation. The resin was packed onto a disposable gravity column (Bio-Rad) and washed with 10 column volumes of wash buffer (40 μM GDN, 300 mM KCl, 2 mM DTT, and 20 mM Tris pH 8.0) and eluted with elute buffer (40 μM GDN, 300 mM KCl, 2 mM DTT, and 20 mM Tris pH 8.0, 40 mM maltose). All purification procedures were carried out on ice or at 4 °C. The eluted HCN1 protein was collected, concentrated, and further purified by size-exclusion chromatography on a Superose 6 column (GE HeathCare) preequilibrated with SEC buffer (40 μM GDN, 300 mM KCl, 2 mM DTT, and 20 mM Tris pH 8.0). Peak fractions were pooled and concentrated to 7.6 mg/ml. All buffers contained protease inhibitors (2 mg/ml leupeptin, 1 mg/ml pepstatin, 50 mg/ml benzamidine, 10 mg/ml aprotinin, and 1 mM AEBSF). Additionally, 1 mM ivabradine was spiked into the protein sample prior to electron microscopy grid preparation.

### Cryo-EM sample preparation and data acquisition

To prepare cryo-EM grids, 3.5 μl of samples were added to 300 Mesh R1.2/1.3 Cu Quantifoil grids (glow discharged at 15 mA for 40 s with a Glow discharge cleaning system). Grids were blotted with qualitative filter paper in a Vitrobot Mark Ⅳ (Thermo Fisher Scientific) at 4 °C and 100% humidity for 3 to 4 s using a blot force of −2 prior to plunging into liquid ethane. For cryo-EM data acquisition, grids were loaded on a Thermo Fisher Scientific 300 kV transmission electron microscope Titan Krios equipped with Gatan K3 direct electron detector. Raw movies were collected using SerialEM in super-resolution mode.

### Imaging processing and 3D reconstruction

Super-resolution image stacks were gain-normalized and imported into cryoSPARC v3.3.2 (https://cryosparc.com/) (42). After motion correction, electron-dose weighting, and contrast transfer function estimation, the initial particle was identified using the cryoSPARC auto picker. Particles were selected and cleaned through several rounds of 2D classification, which were then subjected to *ab initio* reconstruction in C1 using cryoSPARC. The resulting reconstructions were subsequently used as models for heterogeneous refinement in cryoSPARC with all nonjunk particles. The particles that gave a reconstruction with channel features were then 3D classified globally and applied for nonuniform refinement in the class which the amino acid side chains and ivabradine resolution is at its best. To further enhance the resolution, the final particle sets were reextracted with original box size and further applied for final nonuniform refinement and local refinement in C4-symmetry in cryoSPARC, resulting in a density map with overall resolution determined by gold standard Fourier shell correlation using the 0.143 criterion.

### Model building

The initial model was constructed based on the HCN1-apo cryo-EM structure (Protein Data Bank: 5U6O) (25). De novo model building, guided by densities for bulky side chains and disulfide bonds, was conducted using the COOT software (https://www2.mrc-lmb.cam.ac.uk/personal/pemsley/coot/) (43). Subsequent cycles of model building in COOT and real-space refinement using real space refine against the full map in PHENIX (https://phenix-online.org/) were performed to obtain the final refined atomic model (44, 45), which was validated using the MolProbity program (http://molprobity.biochem.duke.edu/) (46), and structural figures were generated using the PyMOL (https://pymol.org/) and UCSF Chimera software (https://www.cgl.ucsf.edu/chimera/) (47).

### Whole cell electrophysiology

Full-length WT or mutant human HCN1 were cloned into the pcDNA3.1 vector for electrophysiological experiments. HeLa cells (from Thermo Fisher Scientific) cultured in Dulbecco's modified Eagle's medium (from Solarbio Life Science) + 10% fetal bovine serum (from ExCell Bio) at 37 °C were transiently transfected using lipofection, and whole-cell patch-clamp recordings were obtained using the Axon 200B amplifier (Molecular Devices) and clampex software (https://www.moleculardevices.com/products/axon-patch-clamp-system/acquisition-and-analysis-software/pclamp-software-suite), 36 to 48 h posttransfection. The extracellular solution contained 110 mM NaCl, 0.5 mM MgCl_2_, 1.8 mM CaCl_2_, 5 mM Hepes, 30 mM KCl, and pH was adjusted to 7.4 with NaOH, while the pipette solution contained 130 mM KCl, 10 mM NaCl, 0.5 mM MgCl_2_, 1 mM EGTA, 5 mM Hepes, and pH was adjusted to 7.4 with KOH. The recordings were performed at room temperature, voltage-clamp mode, and filtered at 1 kHz. The sampling frequency was 10 kHz, and the series resistance was limited to 5 MΩ. To elicit channel currents, step pulses of 3 s duration ranging from −180 mV to −30 mV were applied, followed by a step to −180 mV lasting 1.25 s. The tail currents evoked by the second step were used to determine the voltage-dependent activation curves and calculate the midpoint of activation potential (V_1/2_). The half maximal inhibitory concentration (IC_50_) of drugs was determined by recording currents at a pulse of −140 mV and analyzing current amplitudes at varying drug concentrations. The IC_50_ value was calculated using the Hill equation.

### MD simulation

MD simulations were done using Desmond (https://www.schrodinger.com/platform/products/desmond/), a module of the Schrodinger suite (Schrödinger Release 2021–2, Schrödinger, LLC) to evaluate the stability of the protein-ligand complex (48). The MD simulation system consists of the protein-ligand complex, POPC (1-palmitoyl-2-oleoyl-sn-glycero-3-phosphocholine) lipids, 0.3 M KCl, and simple point charge water molecules using the system builder tool of Desmond. The MD simulation was conducted using default parameters, including recording interval, energy, trajectory, and NPT settings. A total of 1000 frames were recorded and saved to the trajectory during the 200-nsec simulation. A simulation interaction diagram was used for the analysis of the trajectory obtained for the MD simulation.

### Molecular docking

After removing ivabradine from the HCN1-ivabradine complex, the Food and Drug Administration drug library (Selleckchem, Cat #L1300) was docked against HCN1 using Schrödinger Suite 2021-2 (Schrödinger, LLC). The initial small molecule structures were generated and optimized using LigPrep program with the OPLS4 force field, while the protein structure was processed using the default setting within Protein Preparation Wizard with the coordinates of the HCN1-ivabradine complex as input. Molecular docking was performed using the Glide program with high throughput virtual screening (Glide HTVS), the standard-precision (Glide SP) and extra-precision docking method (Glide XP). The top-ranked 50 compounds from XP docking were evaluated further with Prime MM-GBSA studies.

## Data availability

The structures of HCN1-ivabradine complex are available in the following databases:

Cryo-EM density map: EMDB EMD-38961 (https://www.ebi.ac.uk/emdb/EMD-38961).

Atomic coordinates: PDB 8Y60 (https://www.rcsb.org/structure/8Y60).

Additional data related to this article may be requested from the corresponding author.

## Supporting information

This article contains supporting information.

## Conflict of interest

The authors declare that they have no conflicts of interest with the contents of this article.

## Supporting information

Supporting Information

8Y60_HCN1_Ivabradine_NEW.pdb

EMD-38961_HCN1_Ivabradine_NEW.rar
