# Supplementary material for: Structural mechanism of human HCN1 hyperpolarization-activated channel inhibition by ivabradine
Source: J Biol Chem. 2024 Sep 20;300(11):107798. doi: 10.1016/j.jbc.2024.107798 (PMC11530593; doi:10.1016/j.jbc.2024.107798)
Supplement: Supporting Information [file mmc1.docx]

**Supplementary Information**

**Structural mechanism of human HCN1 hyperpolarization-activated channel inhibition by ivabradine**

Tong Che^1,2,†^, Wei Zhang^1,2,†^, Xinyu Cheng^1,2,†^, Sijia Lv^1,2^, Minqing Zhang^1,2^, Yuting Zhang^3^, Tingting Yang^1,2^, Weiwei Nan^3^, Shuangyan Wan^1,2^, Bo Zeng^4,5^, Jian Li^6,^*, Bing Xiong^7,^*, Jin Zhang^1,2,^*

^1^The MOE Basic Research and Innovation Center for the Targeted Therapeutics of Solid Tumors, School of Basic Medical Sciences, Jiangxi Medical College, Nanchang University, Nanchang, Jiangxi 330031, China.

^2^The Second Affiliated Hospital, Jiangxi Medical College, Nanchang University, Nanchang, Jiangxi 330031, China.

^3^Shenzhen Crystalo Biopharmaceutical Co., Ltd, Shenzhen, Guangdong 518118, China

^4^Key Laboratory of Medical Electrophysiology, Ministry of Education and Sichuan Province and Institute of Cardiovascular Research, Southwest Medical University, Luzhou, Sichuan 646000, China.

^5^Department of Endocrinology, Affiliated Hospital of Southwest Medical University, Luzhou, Sichuan 646000, China.

^6^College of Pharmacy, Gannan Medical University, Ganzhou, Jiangxi 341000, China

^7^Department of Medicinal Chemistry, Shanghai Institute of Materia Medica, Chinese Academy of Sciences, 555 Zuchongzhi Road, Shanghai 201203, China

^†^These authors contributed equally to this work.

^*^For correspondence: Jin Zhang ([zhangxiaokong@hotmail.com](mailto:zhangxiaokong@hotmail.com)); Bing Xiong ([bxiong@simm.ac.cn](mailto:bxiong@simm.ac.cn)); Jian Li (rmsl_2040@163.com).

**This PDF file includes:**

Figures S1-7

Table S1


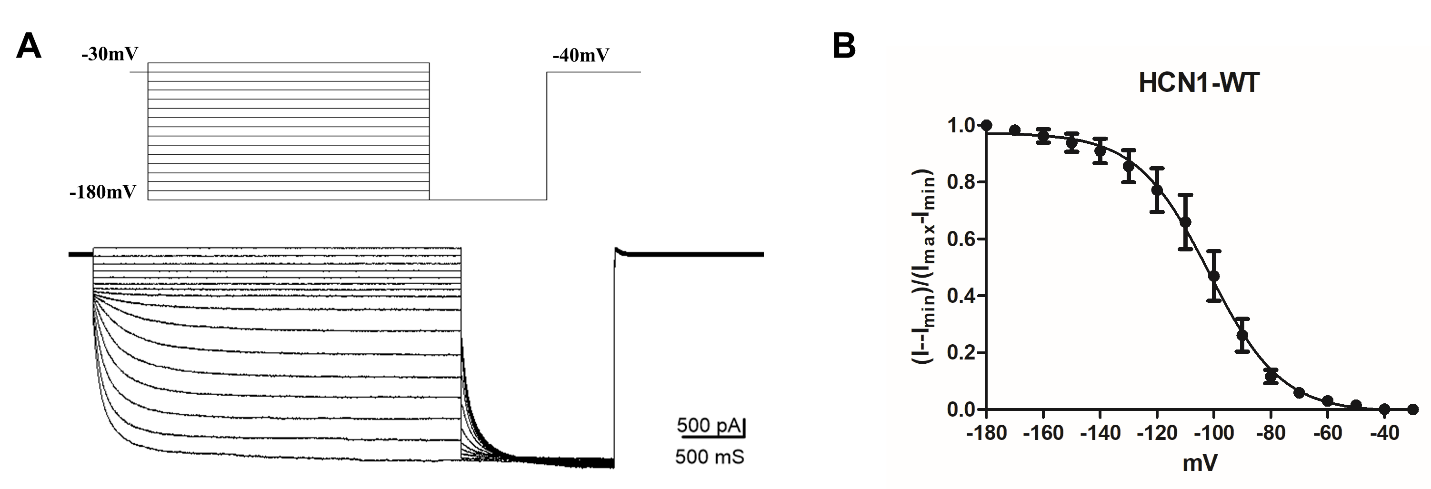


**Figure S1. The electrophysiological characteristics of wild-type hHCN1.** *A,* voltage-dependent activation of HCN1. A representative electrophysiological recording from the Hela cell expressing HCN1 is shown. *B,* activation curves of HCN1. Fraction of the maximum current (n = 6) is plotted against the hyperpolarization voltage and fitted with a single Boltzmann function to obtain the midpoint voltage (V_1/2_).

**
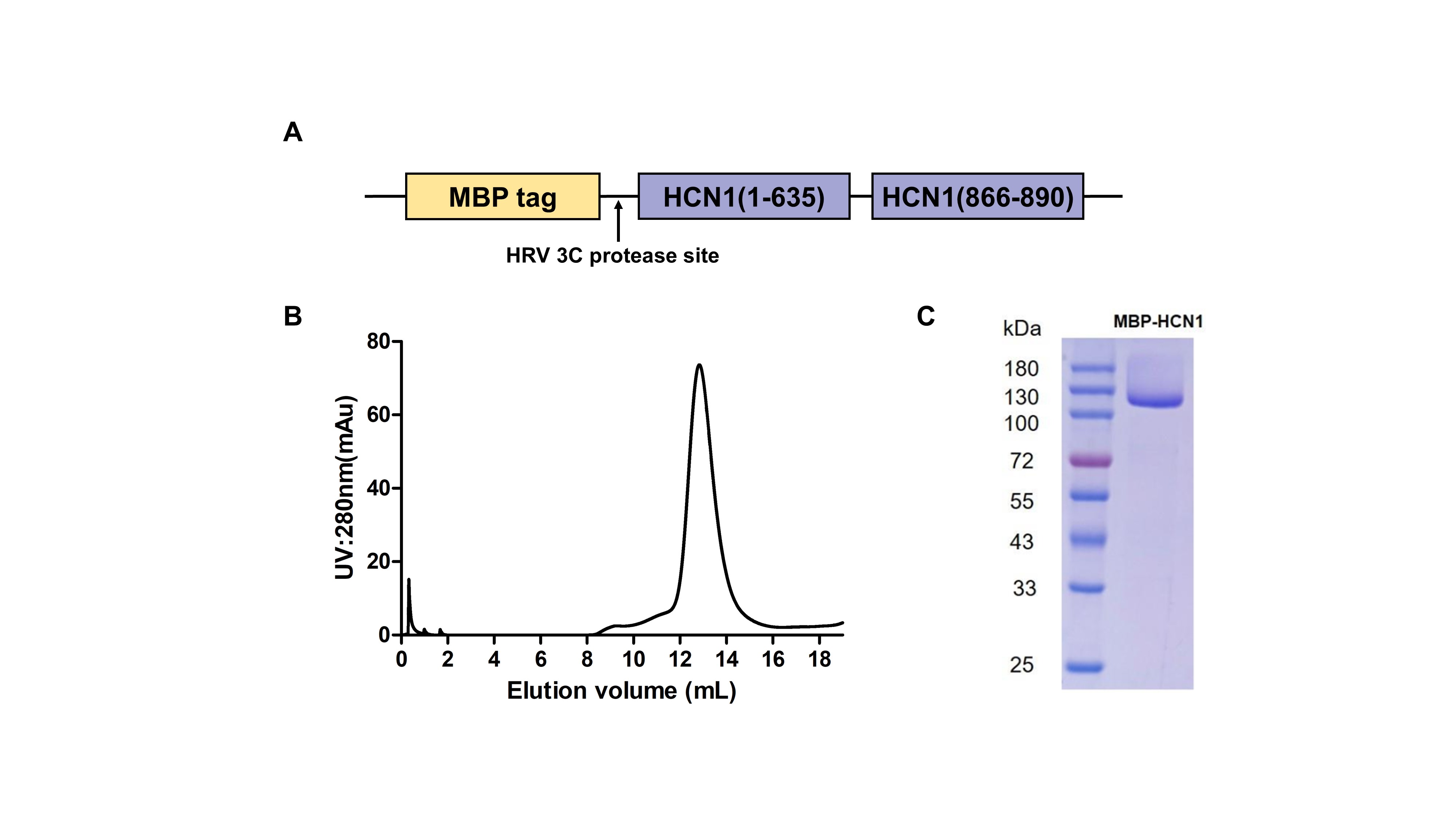
**

**Figure S2. Purification of HCN1.** *A*, a cartoon of the expressed hHCN1 construct. *B,* a representative size-exclusion chromatogram profile of the purified HCN1. *C,* the purified sample of HCN1 was stained by Coomassie blue on SDS-PAGE gel.

**
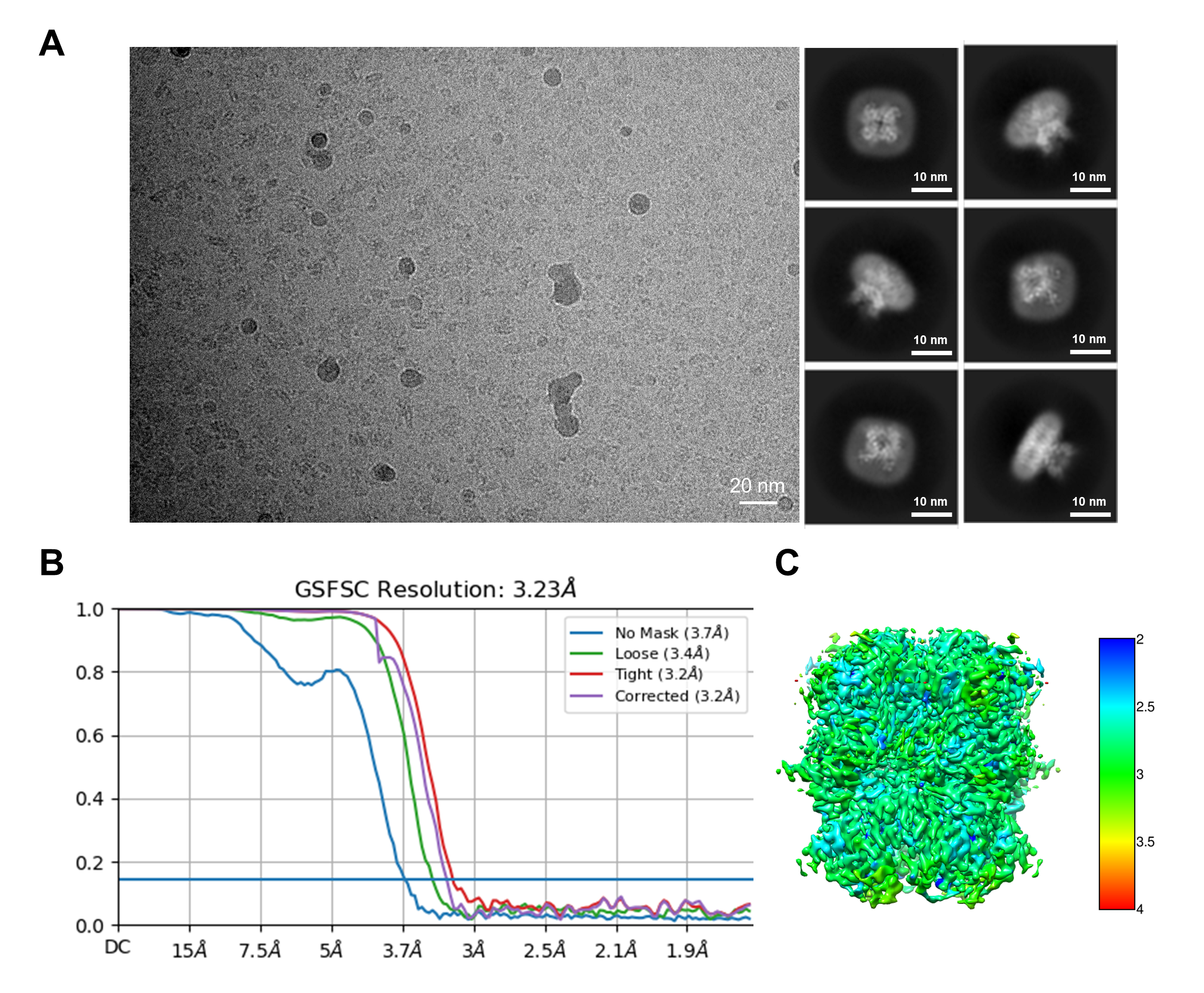
**

**Figure S3. Overview of cryo-EM data for HCN1-ivabradine.** *A,* representative micrograph and 2D class averages. *B,* Gold-Standard Fourier Shell Correlations (GSFSC) curve, the final maps were determined to 3.23 Å. *C,* Local resolution presented as coloring of the HCN1-ivabradine cryo-EM map.

**
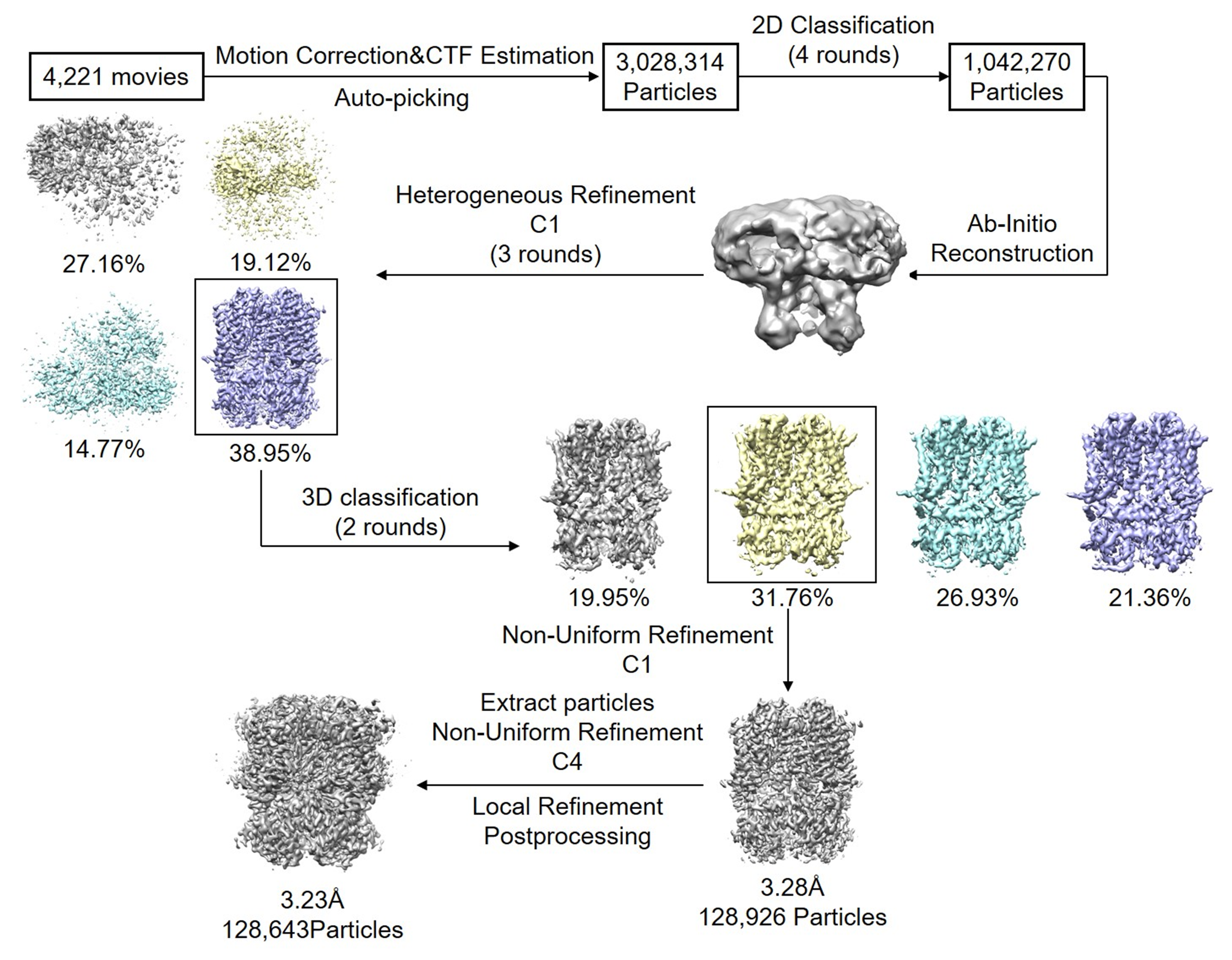
**

**Figure S4. Cryo-EM data processing of HCN1-ivabradine complex.** The workflow of cryo-EM data processing. A total of 4221 movies were collected for HCN1-ivabradine. Particles were autopicked in cryoSPARC, 2D and 3D classifications were conducted to remove bad particles.

**
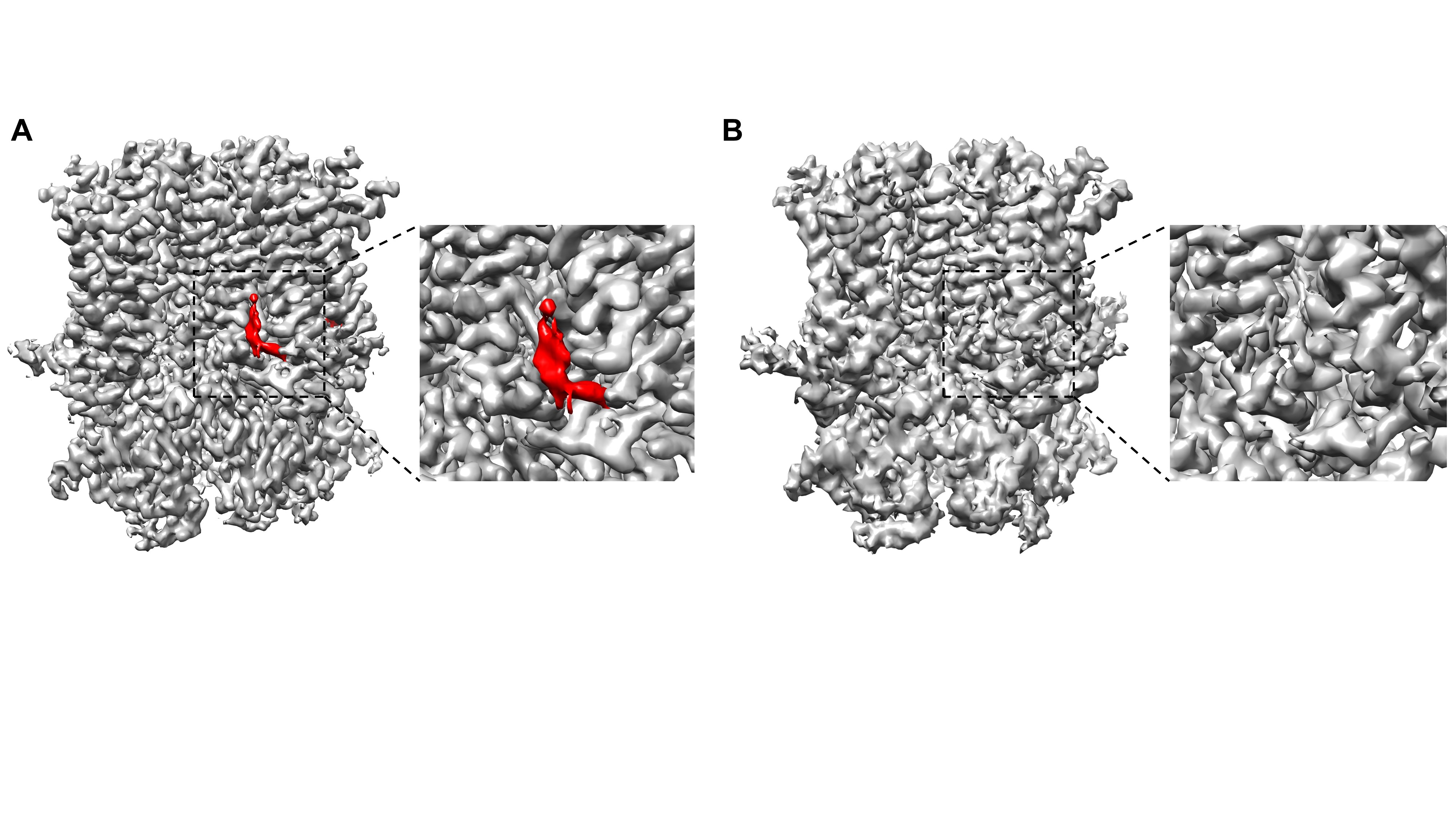
**

**Figure S5. Cryo-EM densities of** **ivabradine binding sites.** *A,* HCN1-ivabradine complex. *B,* HCN1 apo (EMD-8511, PDB: 5U6O). The HCN1 protein is in gray, and the identified ivabradine densities in red.

**
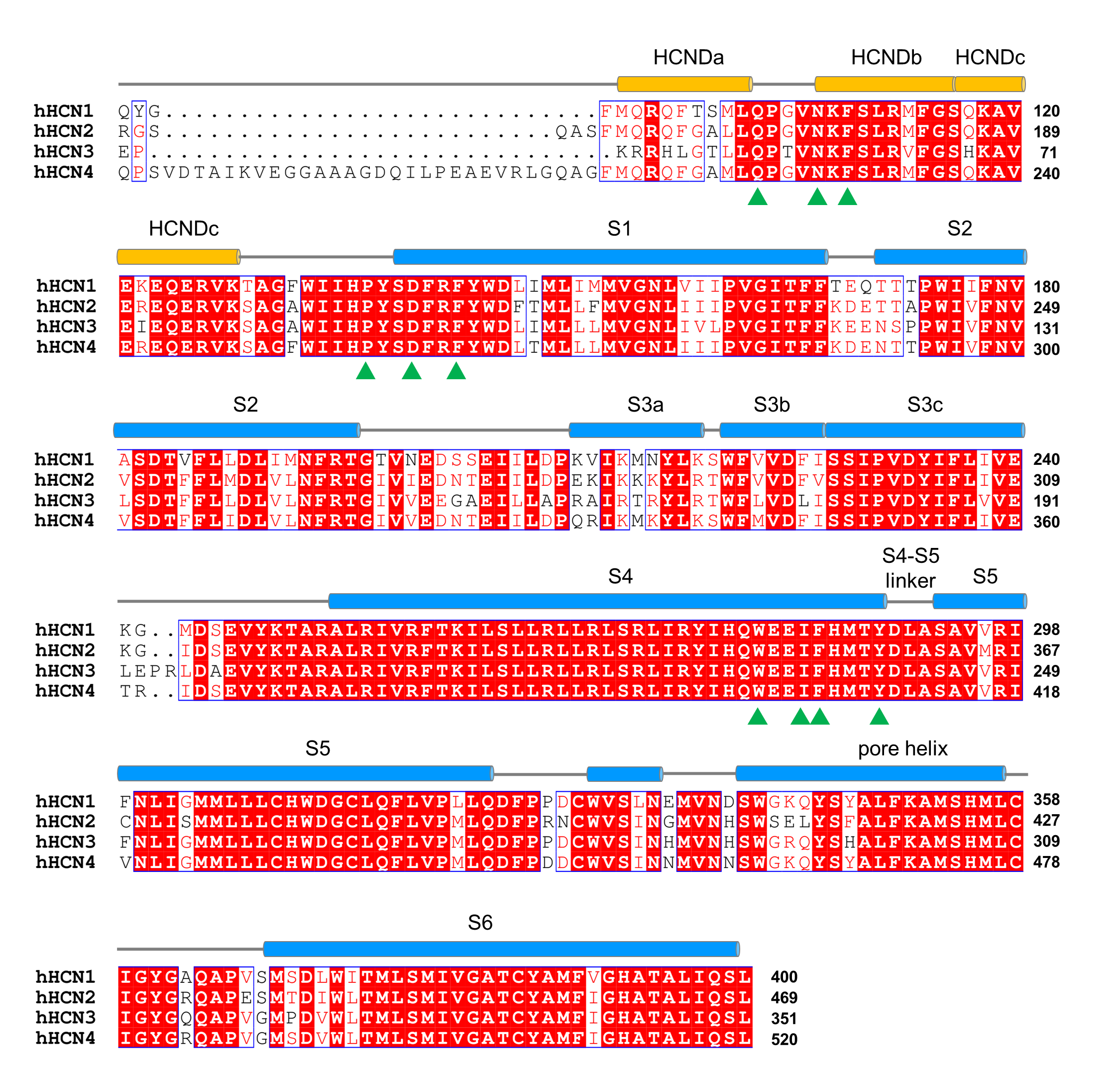
**

**Figure S6. Sequence alignment of human HCN channels.** The TM structured regions of human HCN1-4 (hHCN1-4) are aligned and shown. The identical residues are highlighted in red. The residues interacting with ivabradine are marked with green triangles. Amino acid sequence entries from UniProt databank are: hHCH1, O60741; hHCN2, Q9UL51; hHCN3, Q9P1Z3; hHCN4, Q9Y3Q4.

**
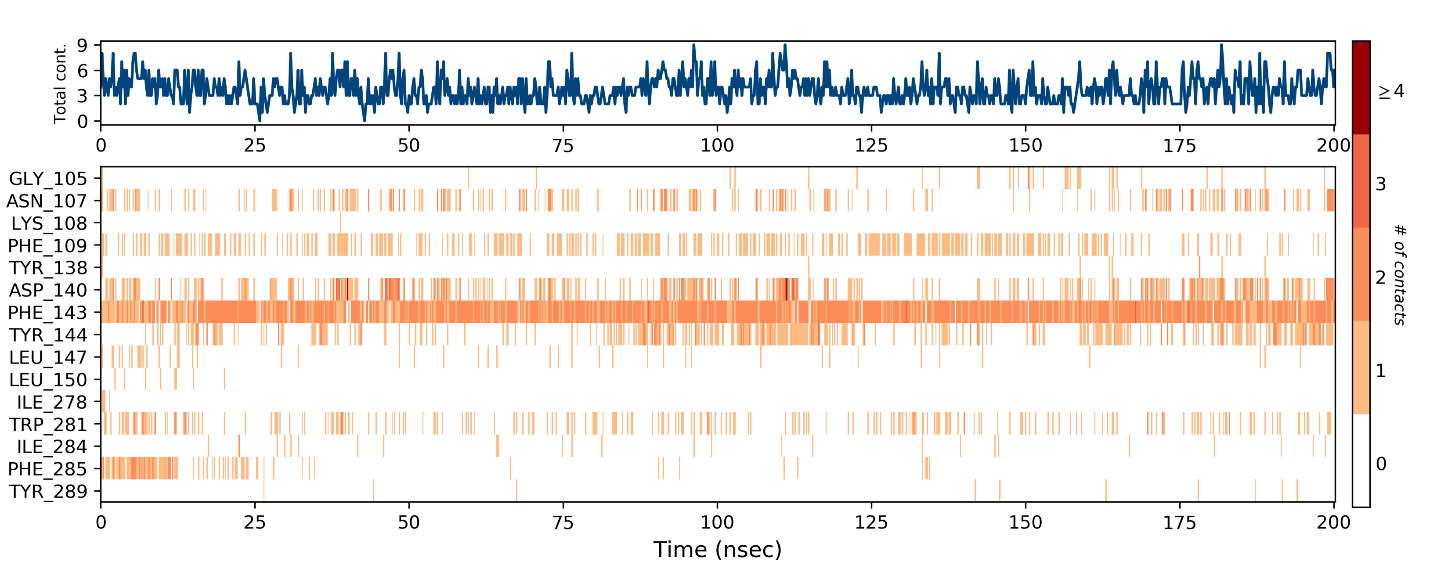
**

**Figure S7. The timeline representation of the interactions and contacts.** The top panel shows the total number of specific contacts the protein makes with the ligand over the course of the trajectory. The bottom panel shows which residues interact with the ligand in each trajectory frame. Some residues make more than one specific contact with the ligand, which is represented by a darker shade of orange, according to the scale to the right of the plot.


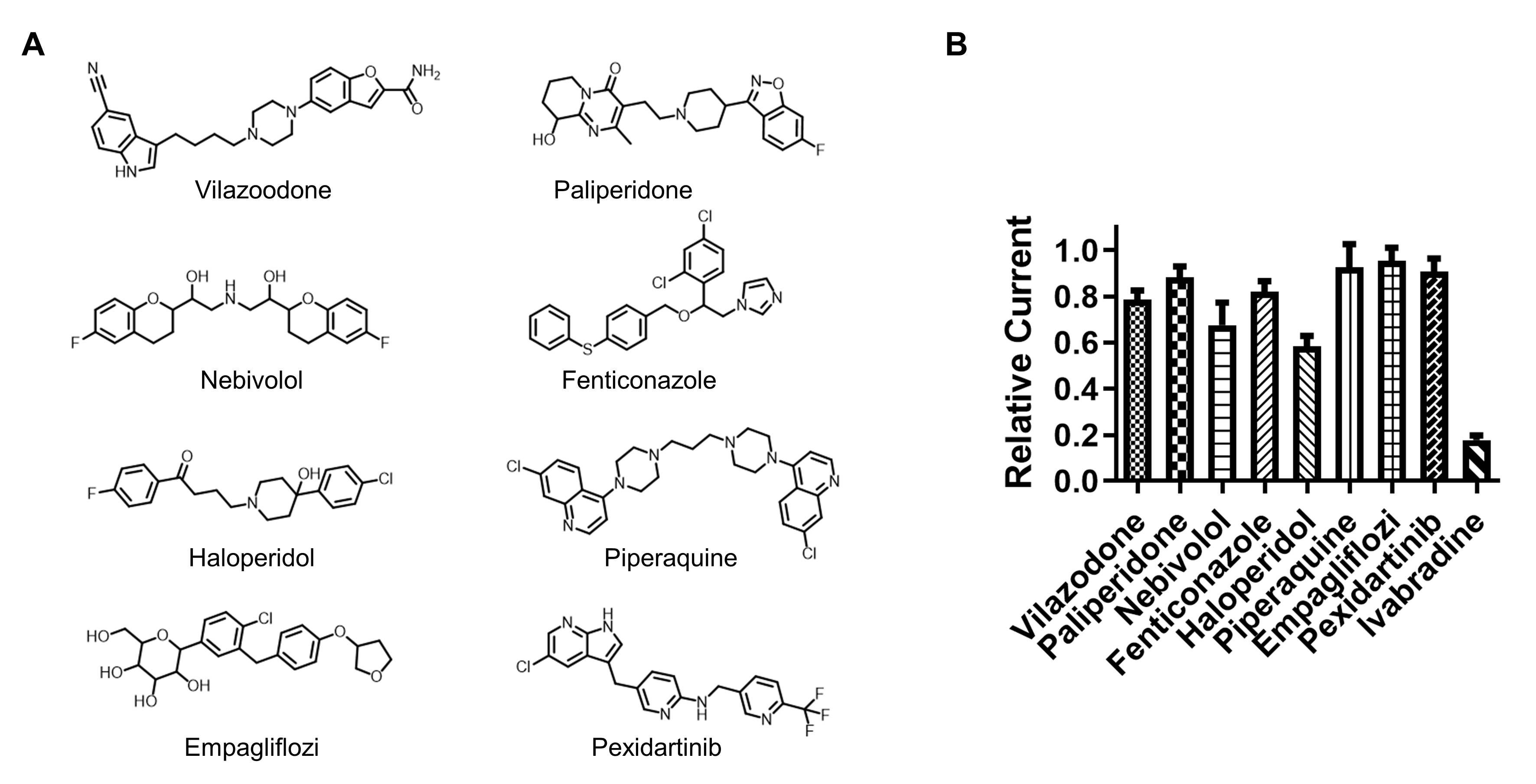


**Figure S8. Chemical structures and activity of selected compounds.** *A,* chemical structures of selected compounds. *B,* relative current of the tested compounds (30 µM) at -140 mV on WT hHCN1 channel.

**Table S1. Cryo-EM data collection, refinement and validation statistics.**

| Structure  EMDB accession code  PDB accession code | HCN1-ivabradine  EMD-38961  8Y60 |
| --- | --- |
| **Data collection and processing** |  |
| Magnification | 105,000 |
| Voltage (kV) | 300 |
| Electron exposure (e–/Å^2^) | 40 |
| Defocus range (μm) | -1.8 ~ -2.2 |
| Pixel size (Å) | 0.83 |
| Symmetry imposed | *C4* |
| Initial particle images (#) | 1,042,270 |
| Final particle images (#) | 128,643 |
| Map resolution (Å)  FSC threshold | 3.23  0.143 |
|  |  |
| **Refinement** |  |
| Initial model used (PDB code) | 5U6O |
| Model resolution (Å)  FSC threshold | 3.5  0.143 |
| **Model composition**  Non-hydrogen atoms  Protein residues  Ligands | 14712  1892  8 |
| ***B* factors (Å^2^)**  Protein  Ligand | 8.95  28.22 |
| r.m.s. deviations  Bond lengths (Å)  Bond angles (°) | 0.01  1.75 |
| **Validation**  MolProbity score  Clashscore  Poor rotamers (%) | 1.76  11.88  0.58% |
| Ramachandran plot  Favored (%)  Allowed (%)  Disallowed (%) | 97.01  2.99  0.00 |
